# Evaluating Diastolic Dysfunction in Children with Congenital Heart Disease: The Role of Stiffness Constant *β*

**DOI:** 10.1016/j.jscai.2025.102576

**Published:** 2025-03-18

**Authors:** Nikhil Thatte, Peter E. Hammer, Gurumurthy Hiremath

**Affiliations:** a Department of Cardiology, Boston Children’s Hospital, Boston, Massachusetts; b Department of Pediatrics, Harvard Medical School, Boston, Massachusetts; c Department of Cardiac Surgery, Boston Children’s Hospital, Boston, Massachusetts; d Department of Surgery, Harvard Medical School, Boston, Massachusetts; e Division of Pediatric Cardiology, Department of Pediatrics, University of Minnesota, Masonic Children’s Hospital, Minneapolis, Minnesota

**Keywords:** diastolic dysfunction, invasive pressure-volume loops, ventricular stiffness constant β

## Background

Diastolic dysfunction (DD) is common in various forms of congenital and acquired heart disease and is challenging to characterize with invasive methods. Two main mechanisms affect diastolic function: abnormalities of lusitropy (active relaxation) and abnormalities of compliance (passive stiffness).^1^ The traditional invasive assessment of DD has focused on its diagnosis using filling pressures rather than on identifying the underlying pathology. Invasive pressure-volume (PV) loops using high-fidelity micromanometer-tipped conductance catheters are valuable for quantifying these diastolic properties in greater detail.^2^^,^^3^

Active relaxation can be characterized using invasive and noninvasive methods by measuring the rate of pressure decay during isovolumetric relaxation, expressed as a pressure half-time *τ* (normal 35 ± 10 milliseconds). A longer *τ* indicates delayed active relaxation and is often seen in patients with heart failure with preserved ejection fraction. Passive compliance, on the contrary, is more challenging to measure.

**Figure 1:**
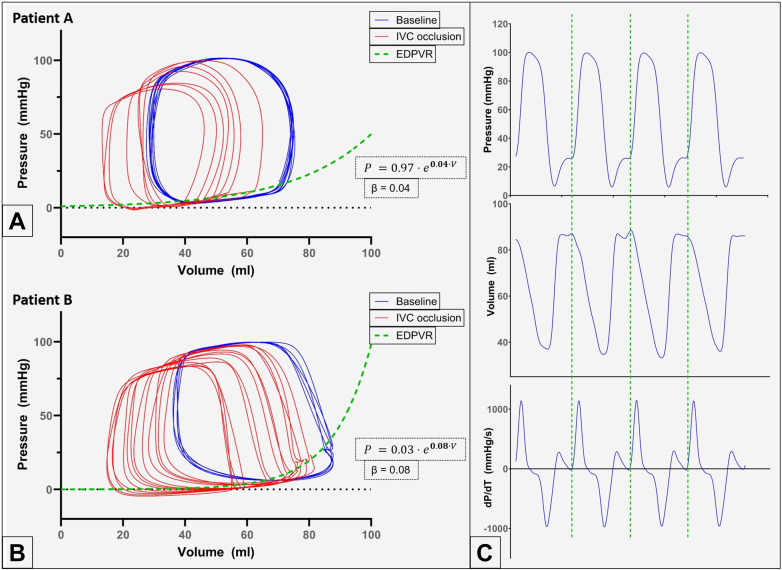
**Pressure-volume (PV) loops from 2 patients showing left ventricular (LV) compliance curves obtained after recording PV loops at baseline and following temporary occlusion of the inferior vena cava.** (A) Patient A demonstrates mild LV diastolic dysfunction (DD) with the ventricle operating on the flatter portion of the compliance curve. (B) Patient B demonstrates severe LV DD with the ventricle operating on the steep portion of the compliance curve. See text for details of clinical presentation and interpretation of compliance curve. (C) Simultaneous visualization of the pressure vs time (top row), volume vs time (middle row), and dP/dT vs time (bottom row) traces for patient B. The location of end-diastole (dashed green lines) can be identified as the location at which there is a sudden steep rise in pressure and dP/dT, which marks the onset of the isovolumetric contraction phase of systole. This point also typically corresponds to the location of the peak of the R wave on the electrocardiogram. The volume vs time curves show a plateau in the second half of diastole. Most of the ventricular filling occurs in early diastole. EDPVR, end-diastolic pressure-volume relationship; IVC, inferior vena cava.

The passive compliance component of diastolic function is most accurately assessed by characterizing the ventricular end-diastolic pressure-volume relationship (EDPVR).^4^ This involves measuring PV loops at baseline and changing loading conditions, which can be accomplished by temporarily occluding the inferior vena cava with a soft balloon. In select cases, an increase in preload can be achieved by a rapid infusion of crystalloid or test occlusion of an atrial septal defect in those with left-to-right atrial shunts (increases left ventricular [LV] preload). This loading change combination allows recording a family of PV loops, as shown in Figure 1. The curve obtained by connecting the end-diastolic PV points of this family of loops is the EDPVR, which can then be used to characterize the diastolic properties of the ventricle. The stiffness constant *β* is an index of chamber stiffness and is elevated in DD due to abnormal compliance.^4^^,^^5^ Abnormalities in compliance are important contributors to DD in congenital heart disease.^5^, ^6^, ^7^, ^8^

## Chamber stiffness constant β

Chamber stiffness is the slope of the EDPVR, the change in ventricular pressure relative to a change in volume (dP/dV).^4^ Unlike the linear end-systolic PV relationship, the EDPVR is intrinsically nonlinear and requires a unique mathematical approach to characterize its properties. A commonly used approach involves an exponential curve fit of the end-diastolic PV points, yielding a curve defined by the equation $P=k\cdot e^{\beta V}$ (where *P* = end-diastolic pressure, *k* = initial value when volume = 0, β= exponential curve fitting constant, and *V* = end-diastolic volume).^4^ Owing to the mathematical properties of this relation, the value of *β* varies with the size of the ventricle, introducing an added challenge for pediatric patients undergoing normal growth. To address the challenge of comparing pediatric patients across widely varied body and LV sizes, a proposed approach is to index the value of *β* to the ventricular wall volume (*V*_w_), which can be determined from cardiac magnetic resonance imaging (MRI).^4^ This converts *β* (which has units of mL^−^^1^) to a dimensionless constant *β*_w_ via the following equation: $\beta_{w}=\beta \cdot V_{w}$. Patients with a higher *β*, or a higher *β*_w_, have a stiffer ventricle. Normal values for *β* were reported by Zile et al^1^ in a study of adults with heart failure with preserved ejection fraction that included 10 healthy controls (*β* for controls measured 0.01 ± 0.01 mL^−^^1^); a *β* ≥ 0.015 mL^−^^1^ is considered abnormal.^9^^,^^10^ However, large data sets for normal values, particularly in pediatrics, are lacking. Stiffness constant *β* and *β*_w_ are potentially valuable tools in evaluating and quantifying DD in children.

## Case examples

Patient A is a 6-year-old boy with a history of hypoplastic left heart syndrome (mitral stenosis and aortic stenosis variant) who initially underwent a traditional single-ventricle palliation to a bidirectional Glenn shunt. At 2.5 years of age, he was referred for consideration of biventricular conversion.^11^ Accordingly, a left modified Blalock-Taussig-Thomas shunt was placed with a separation created between the right bidirectional Glenn shunt and the left Blalock-Taussig-Thomas shunt, and the atrial septum was restricted to volume load the LV. Subsequently, he underwent complete biventricular repair at 5 years of age. On the most recent MRI, he had normal mitral and aortic valve function, and no significant endocardial fibroelastosis (EFE) was noted. Cardiac catheterization with PV loop analysis was performed as part of institutional protocol for postoperative surveillance of patients undergoing biventricular conversion after initial single-ventricle palliation for hypoplastic left heart syndrome. Baseline EDP was elevated at 15 mm Hg. The patient was asymptomatic from a cardiac standpoint but exhibited evidence of mild LV DD related to impaired passive compliance with normal active relaxation; *β* was elevated at 0.04 mL^−^^1^ and *β*_w_ measured 1.49; *τ* was normal at 28 milliseconds (Figure 1A, Supplemental Table S1). The LV operates on the flat portion of the compliance curve, and small changes in volume lead to small changes in pressure.

Patient B is a 15-year-old boy who was prenatally diagnosed with severe valvar aortic stenosis and underwent fetal transuterine transthoracic aortic balloon valvuloplasty. Postnatally, the left ventricle was felt to be inadequate to support the systemic circulation, so he was palliated in the traditional 3-stage fashion to a single-ventricle Fontan circulation by 3 years of age. Owing to promising growth in the left ventricle and a desire to avoid the long-term complications of the Fontan circulation, he underwent staged conversion to a biventricular repair at 10 years of age and, most recently, a Ross procedure that was completed at 14 years of age. A cardiac MRI showed the persistence of LV EFE. Catheterization demonstrated severely elevated LV EDP at 28 mm Hg. PV loops demonstrated a sharp rise in filling pressure in the terminal stage of diastole (corresponding to atrial contraction) with minimal volume change (Figure 1B, Supplemental Table S1). The correct position of the end-diastolic PV point on the PV loop was determined using simultaneous visualization of the pressure-time, volume-time, and dP/dT-time traces (Figure 1C); *β* was severely elevated at 0.08 mL^−^^1^ and *β*_w_ measured 4.17. Notably, *τ* was normal at 31 milliseconds. The patient was significantly limited in exercise capacity and was referred to the heart failure team. This patient demonstrated significant LV DD related to abnormal passive compliance, characterized by severely elevated *β* and *β*_w_, with normal active relaxation (*τ*). The left ventricle is operating on the steep portion of the compliance curve (Figure 1B), and small changes in volume lead to large changes in EDP. The patient was treated with an aggressive oral heart failure regimen, including diuretics, and a follow-up catheterization 6 months later showed a reduced EDP of 17 mm Hg.

## Conclusion

LV DD is challenging to characterize. The pressure half-time *τ* and the ventricular stiffness constant *β* (or the indexed *β*_w_), are useful markers to characterize the mechanism of LV DD accurately as impairment of active relaxation or passive compliance, respectively. Additional studies are needed to explore the underlying mechanisms of DD in different patient populations. Studies of DD should focus on distinguishing between impairment of active relaxation and passive compliance at a phenomenologic level, identifying the molecular pathways that lead to increased myofibril stiffness, and determining the specific role of EFE in causing DD in patients with congenital heart disease at a mechanistic level. This understanding could help identify potential treatment targets and develop effective therapies for this complex condition. Readers are referred to Burkhoff et al^4^ for a more in-depth review of invasive analysis of LV diastolic function.

## Pearls in Hemodynamics

• Diastolic dysfunction results from abnormalities in active relaxation (lusitropy) or abnormalities of passive filling (compliance), the latter being more common in congenital heart disease.
• The passive filling component of diastolic function is best assessed by characterizing the end-diastolic pressure-volume relationship through pressure-volume loop analysis under varying loading conditions
• The ventricular stiffness constant *β* (or the indexed *β*_*w*_), along with the pressure half-time *τ*, is a valuable marker to characterize the left ventricular diastolic dysfunction mechanism accurately.

## Footnotes

To access the supplementary material accompanying this article, visit the online version of the *Journal of the Society for Cardiovascular Angiography & Interventions* at 10.1016/j.jscai.2025.102576.
