# Supplementary material for: Evaluating Diastolic Dysfunction in Children with Congenital Heart Disease: The Role of Stiffness Constant β
Source: J Soc Cardiovasc Angiogr Interv. 2025 Mar 18;4(3Part A):102576. doi: 10.1016/j.jscai.2025.102576 (PMC11993888; doi:10.1016/j.jscai.2025.102576)
Supplement: Supplemental Table S1 [file mmc1.docx]

**Supplemental Table 1:** Summary of invasive indices of diastolic function in the two patients.

| Parameter | Patient A | Patient B |
| --- | --- | --- |
| Tau (τ) | 28 msec | 31 msec |
| LV EDP | 15 mmHg | 28 mmHg |
| Stiffness constant *β* | 0.04 ml^-1^ | 0.08 ml^-1^ |
| Indexed stiffness constant $\boldsymbol{\beta}_{\boldsymbol{w}}$ | 1.49 | 4.17 |

LV EDP = left ventricular end-diastolic pressure
